# Risk factors for in-patient admission among adults with intellectual disability and autism: investigation of electronic clinical records

**DOI:** 10.1192/bjo.2020.135

**Published:** 2020-12-01

**Authors:** Rory Sheehan, Jennifer Mutch, Louise Marston, David Osborn, Angela Hassiotis

**Affiliations:** Division of Psychiatry, University College London, UK; Community Learning Disability Service, Lynebank Hospital, Scotland, UK; Primary Care and Population Health, University College London, UK; Division of Psychiatry, University College London, UK; Division of Psychiatry, University College London, UK

**Keywords:** Admissions, autism, intellectual disability, crisis care, risk

## Abstract

Adults with intellectual disability or autism are at risk of psychiatric admission which carries personal, social and economic costs. We identified 654 adults with intellectual disability or autism in the electronic clinical records of one mental health trust. We investigated the demographic and clinical factors associated with admission and readmission after discharge. Young male patients with intellectual disability, schizophrenia and previous admissions are most at risk of the former, whereas affective and personality disorders predict the latter. Both community intellectual disability services and mental health crisis care must focus on providing effective support for those patients.

Both intellectual disability and autism are associated with significant additional psychiatric morbidity.^1,2^ Poor understanding of mental disorder in these groups, the presence of challenging behaviour, and lack of access to appropriate community-based care and support may lead to unnecessary or prolonged admissions to psychiatric in-patient care. Reducing inappropriate psychiatric admissions is a cornerstone of current UK healthcare policy, and a priority for people with intellectual disability, autism and their social networks.^3^

NHS England mandate that clinical commissioning groups maintain ‘at risk of admission’ registers that identify those at greatest risk of hospital admission and ensure these individuals are prioritised and supported effectively at times of crisis.^4^ Indicators for inclusion on the register are not well established and vary locally.

We used patient-level data to explore associations with in-patient admission, and with readmission following discharge, of adults with intellectual disability and/or autism accessing care from a mental health trust.

## Method

### Data source

Data were obtained from the Camden and Islington NHS Foundation Trust electronic clinical record, using the Clinical Record Interactive Search (CRIS) tool. The Trust provides services to an ethnically and socioeconomically diverse population of approximately 500 000 people. Their electronic clinical record contains anonymised information of over 130 000 adults, and the Trust only admits mentally ill individuals who reside within its area boundaries and only those with mild-to-moderate intellectual disability.^5,6^ Data can be extracted from both structured fields and from the free-text records, applying natural language processing (NLP) algorithms. The authors assert that all procedures contributing to this work comply with the ethical standards of the relevant national and institutional committees on human experimentation and with the Helsinki Declaration of 1975, as revised in 2008. All procedures involving human subjects/patients were approved by the East of England-Cambridge Central Research Ethics Committee (approval number 14/EE/0177). The specific project was approved by the Camden and Islington CRIS oversight panel.

### Study cohort

Adults (≥18 years) with ICD-10 diagnostic codes F70–F79 (mental retardation) and/or F84 (pervasive developmental disorders) who had accessed secondary mental healthcare within the cohort period were included. In addition, NLP was used to explore the clinical records (assessment and progress notes) for terms related to intellectual disability and/or autism. Eligibility for inclusion of those identified via NLP was validated by one of the authors (J.M.), with queries resolved by discussion with another author (A.H.). Entry to the cohort was 1 January 2009, the date of turning 18 years or discharge from hospital (if in hospital on 1 January 2009), whichever was latest. Exit from the cohort was 30 November 2018, date of death or admission to hospital (if in hospital on 30 November 2018), whichever was earliest.

### Outcomes and covariates

The main outcome was first admission to psychiatric in-patient care during the cohort period. The secondary outcome was readmission to the acute care pathway (psychiatric in-patient care or crisis team intervention) within 12 months following discharge from the first admission in the cohort period.

We extracted data on diagnostic group (intellectual disability with or without autism, and autism only); age at cohort entry; gender; ethnicity; diagnosis of mental illness (coded according to the ICD-10^7^); social deprivation (estimated with the Index of Multiple Deprivation^8^); and presence of internalising or externalising challenging behaviour (measured with the Health of The Nation Outcome Scales^9^) for each individual.

### Statistical analysis

All categorical variables were analysed with chi-squared tests, apart from eating disorders, which was analysed with Fisher's exact test because of the small number in the cells that violated the test assumptions. Continuous variables were analysed with *t*-tests, with the exception of median duration of admission, which was analysed with the Mann–Whitney *U*-test because of skewness. Logistic regression was used to explore associations between in-patient admission and demographic and clinical variables and discharge to a destination other than a person's usual residence. We plotted time to readmission to the acute care pathway within 12 months of discharge, using a Kaplan–Meier plot, and utilised Cox regression to determine differences in time to readmission between the groups controlling for confounders. We carried out multiple imputation using chained equations for missing data for the analyses related to first admission. We calculated the rate of in-patient admissions during each year of the cohort and tested the change in the rate over time by using Poisson regression, with person-years as the exposure. Data were analysed with Stata version 16 for Windows.

## Results

### Profile of people using services

We identified 339 adults with intellectual disability with or without autism and 315 adults with autism only who accessed mental healthcare services between January 2009 and November 2018. Each person contributed an average of 7.94 years follow-up.

The autism only group were younger and included a greater proportion of males than the intellectual disability group. Adults with intellectual disability were more likely to have been diagnosed with schizophrenia spectrum disorders (47.8% *v*. 18.1%, *P* < 0.001), whereas a greater proportion in the autism-only group had a diagnosis of anxiety disorder (11.2% *v*. 24.9%, *P* < 0.001) (Table 1). Approximately a third of the total sample were admitted to hospital at least once during the cohort period.

**Table 1:** Cohort summary

Variable	Intellectual disability with/without autism (*n* = 339)	Autism only (*n* = 315)	Total sample (*n* = 654)	*P*-value
Degree of intellectual disability				
Mild	114	–	114	
Moderate-to-severe	16	–	16	
Missing/not recorded	209	–	209	
Not applicable	–	315	315	
Gender				
Male	177 (52.2%)	235 (74.6%)	412 (63.0%)	<0.001
Female	162 (47.8%)	80 (25.4%)	242 (37.0%)	
Age at cohort entry, mean (s.d.)	35.8 (14.9)	30.5 (13.27)	33.2 (14.4)	<0.001
Ethnic group				
White	205 (60.5%)	171 (54.3%)	376 (57.5%)	0.226
Not White	112 (33.0%)	75 (23.8%)	187 (28.6%)	
Missing	22 (6.5%)	69 (21.9%)	91 (13.9%)	
Index multiple deprivation, mean (s.d.)	33.2 (12.37)	31.2 (11.49)	32.3 (12.00)	
Missing	19	24	43	0.016
Died in cohort period				
Yes	20 (5.9%)	6 (1.9%)	26 (4.0%)	0.009
No	319 (94.1%)	309 (98.1%)	628 (96.0%)	
Admitted in cohort period				
Yes	155 (45.7%)	61 (19.4%)	216 (33.0%)	<0.001
No	184 (54.3%)	254 (80.6%)	438 (67.0%)	
Crisis team contact in cohort period				
Yes	151 (44.5%)	108 (34.3%)	259 (39.6%)	<0.001
No	188 (55.5%)	207 (65.7%)	395 (60.4%)	
HoNOS externalising				
No problem	132 (38.9%)	167 (53.0%)	299 (45.7%)	<0.001
Problem	109 (32.2%)	65 (20.6%)	174 (26.6%)	
Missing	98 (28.9%)	83 (26.3%)	181 (27.7%)	
HoNOS internalising				
No problem	204 (60.2%)	186 (59.0%)	390 (59.6%)	0.201
Problem	37 (10.9%)	46 (14.6%)	83 (12.7%)	
Missing	98 (28.9%)	83 (26.3%)	181 (27.7%)	
Diagnosis				
F00–F09 (dementia)	22 (6.5%)	8 (2.5%)	30 (4.6%)	0.014
F10–F19 (drug/alcohol)	37 (14.7%)	15 (5.4%)	52 (9.9%)	<0.001
F20–F29 (SSD)	120 (47.8%)	50 (18.1%)	170 (32.2%)	<0.001
F30–F39 (mood)	57 (22.7%)	57 (20.6%)	114 (21.6%)	0.552
F40–F48 (anxiety)	28 (11.2%)	69 (24.9%)	97 (18.4%)	<0.001
F50–F59 (eating disorders)	2 (0.8%)	4 (1.4%)	6 (1.1%)	0.690
F60–F60 (personality disorder)	39 (15.5%)	37 (13.4%)	76 (14.4%)	0.498
F90–F98 (childhood onset)	11 (4.4%)	29 (10.5%)	40 (7.6%)	0.008
Missing any diagnosis	138 (40.7%)	120 (38.1%)	258 (39.4%)	
Antipsychotic (ever prescribed)				
Yes	230 (67.9%)	112 (35.6%)	312 (47.7%)	<0.001
No	109 (32.2%)	203 (64.4%)	342 (52.3%)	
Other psychotropic (ever prescribed)				
Yes	254 (74.9%)	181 (57.5%)	435 (66.5%)	<0.001
No	85 (25.1%)	134 (42.5%)	219 (33.5%)	
Admitted to hospital before cohort entry				
Yes	65 (19.2%)	25 (7.9%)	90 (13.8%)	<0.001
No	274 (80.8%)	290 (92.1%)	564 (86.2%)	
Cohort contribution, years, mean (s.d.)	8.28 (1.53)	7.57 (2.30)	7.94 (1.97)	<0.001

HoNOS, Health of The Nation Outcome Scales; SSD, schizophrenia spectrum disorders.

### Admission and discharge within the cohort period

The median duration of first admission in the cohort period was 34 days (interquartile range, 11–95 days) in the intellectual disability group, and 27 days (interquartile range, 14–58 days) in the autism-only group (*P* = 0.211). Likelihood of psychiatric admission was higher in those with intellectual disability (adjusted odds ratio (aOR), 4.00; 95% CI 2.41–6.63), males (aOR, 2.28; 95% CI 1.39–3.75), younger age (aOR, 0.98; 95% CI 0.97–1.00) and in those with a diagnosis of schizophrenia spectrum disorder (aOR, 5.08; 95% CI 3.00–8.61), affective disorder (aOR, 2.23; 95% CI 1.29–3.83), personality disorder (aOR, 1.94; 95% CI 1.02–3.68) and record of a previous admission (aOR, 2.18; 95% CI 1.17–4.05).

Discharge destination after first admission was usual residence for 57.4% of adults with intellectual disability and 78.7% for the autism-only group. Discharge to a place other than usual residence was associated with longer duration of admission (aOR, 1.01; 95% CI 1.00–1.01), and having externalising or internalising challenging behaviours (aOR, 3.34; 95% CI 0.65–17.13 and aOR, 9.87; 95% CI 1.75–55.70, respectively). Living in a more deprived area was associated with discharge to usual residence (aOR, 0.90; 95% CI 0.83–0.97).

There was no statistically significant change in the rate of admissions over time, including the years 2014–2018, during which the new policy initiative ‘Transforming Care’ was implemented in England.^10^

### Re-entry to the acute care pathway

A total of 40% of those who had an admission during the cohort period were readmitted or had contact with mental health crisis services within 12 months of discharge, mostly within the first 6 months after discharge (see Supplementary Fig. 1 available at https://doi.org/10.1192/bjo.2020.135). Those with autism only had a greater risk of readmission, although this difference was not statistically significant (aOR, 1.43; 95% CI 0.66–3.05). Comorbid diagnoses of affective disorder or personality disorder were significantly associated with readmission (aOR, 3.11; 95% CI 1.34–7.23 and aOR, 8.28; 95% CI 2.85–24.04, respectively).

## Discussion

In summary, we found that young male patients with intellectual disability, schizophrenia spectrum disorder and previous admissions are most at risk of a new admission to psychiatric in-patient care, whereas only affective and personality disorders predict readmission. Almost half of those with an intellectual disability are unable to return to their original accommodation following discharge. Both adults with intellectual disabilities or autism only are vulnerable to admission, but each group may have differential needs and both are likely to be in contact with acute mental health services soon after discharge. The reasons behind the pattern of readmissions following contacts with crisis teams merits further exploration. It may be that patient characteristics may affect outcome, or that potentially patients are discharged with a degree of unmet need or that their needs are not fully understood at the point of contact with crisis professionals. As community intellectual disability services are required to maintain a dynamic register of patients ‘at risk of admission’, our findings can inform the effective use of the register by guiding the provision of services directed to those patients. These must include proactive liaison with crisis care or home treatment teams, and availability of staff with experience in working with those individuals at the time of assessment.

Being discharged to a place other than their usual residence demonstrates the potential for in-patient admission to disrupt a person's life course. Both internalising and externalising behaviours were strongly associated with the original residence being deemed unsuitable to support the person following discharge in about half of adults with intellectual disability. This finding underlines the need for responsive specialist behaviour community services for patients with complex presentations.

The cohort period includes the years spanning the innovations introduced as part of Transforming Care, including the introduction of Care and Treatment Reviews.^10^ It would appear that the initiation of such reviews has not influenced the rate of admissions of people with intellectual disability or with autism to psychiatric in-patient wards, at least in this area. This is in accord with NHS Digital data, which show that Transforming Care has not had the desired effect on reduction of admissions.^11^

This is the first study using routinely collected clinical adult mental health data to investigate the pattern of admissions of people with intellectual disability and with autism. The results are highly relevant in the context of changes in the care of this population group, which began with the launch of the White Paper ‘Valuing People’^12^ in 2001. Valuing People promoted the use of general psychiatric services by people with intellectual disability and with autism who have mental ill health, but its implementation has not been fully appraised, and this group continue to experience health inequalities related to poor access to healthcare, which may extend to inappropriate in-patient admission.

The study also has limitations, including lack of inclusion of those people who were transferred to out-of-area facilities, for example, specialist hospitals. Psychiatric comorbidities could be attributed to reverse causality as those who are admitted are more likely to receive a mental health diagnosis because of increased contact.^13^

In conclusion, the development and maintenance of ‘at risk of admission’ registers with monthly reporting by clinical commissioning groups to NHS England ensures close scrutiny of outcomes for people with intellectual disability and/or autism who are acutely mentally ill. In order for the registers to be most effectively used, they should be accompanied by robust and clearly defined responses by community intellectual disability and crisis care teams, tailored to the most vulnerable patients. Despite early UK studies that failed to show efficacy,^14^ research outside the UK suggests that assertive outreach models may be adapted successfully for this population.^15^ Given how much is at stake in terms of poor quality of life and years spent in in-patient units, revisiting prevention of admissions and emergency care response is urgently needed.

## Data Availability

This database of anonymised data is accessible only to approved researchers within Camden and Islington Foundation NHS Trust and its partner research organisations.

## Supplementary material

For supplementary material accompanying this paper visit http://dx.doi.org/10.1192/bjo.2020.135. click here to view supplementary material
