# Supplementary material for: Risk factors for in-patient admission among adults with intellectual disability and autism: investigation of electronic clinical records
Source: BJPsych Open. 2020 Dec 1;7(1):e5. doi: 10.1192/bjo.2020.135 (PMC7791557; doi:10.1192/bjo.2020.135)

**Table 1 Cohort summary**

| **Variable** | **ID*±Autism (*n*=339)** | **Autism only (*n*=315)** | **Total sample (*n*=654)** | ***p*-value** |
| --- | --- | --- | --- | --- |
| **Degree of ID**  Mild  Moderate-Severe  Missing/not recorded  N/A | 114  16  209  - | -  -  -  315 | 114  16  209  315 |  |
| **Sex**  Male  Female | 177 (52.2%)  162 (47.8%) | 235 (74.6%)  80 (25.4%) | 412 (63.0%)  242 (37.0%) | <0.001 |
| **Age at cohort entry** (mean, SD) | 35.8 (14.9) | 30.5 (13.27) | 33.2 (14.4) | <0.001 |
| **Ethnic group**  White  Not White  Missing | 205 (60.5%)  112 (33.0%)  22 (6.5%) | 171 (54.3%)  75 (23.8%)  69 (21.9%) | 376 (57.5%)  187 (28.6%)  91 (13.9%) | 0.226 |
| **Index multiple deprivation** (mean, SD)  Missing | 33.2 (12.37)  19 | 31.2 (11.49)  24 | 32.3 (12.00)  43 | 0.016 |
| **Died in cohort period**  Yes  No | 20 (5.9%)  319 (94.1%) | 6 (1.9%)  309 (98.1%) | 26 (4.0%)  628 (96.0%) | 0.009 |
| **Admitted in cohort period**  Yes  No | 155 (45.7%)  184 (54.3%) | 61 (19.4%)  254 (80.6%) | 216 (33.0%)  438 (67.0%) | <0.001 |
| **Crisis team contact in cohort period**  Yes  No | 151 (44.5%)  188 (55.5%) | 108 (34.3%)  207 (65.7%) | 259 (39.6%)  395 (60.4%) | <0.001 |
| **HoNOS externalising**  No problem  Problem  Missing | 132 (38.9%)  109 (32.2%)  98 (28.9%) | 167 (53.0%)  65 (20.6%)  83 (26.3%) | 299 (45.7%)  174 (26.6%)  181 (27.7%) | <0.001 |
| **HoNOS internalising**  No problem  Problem  Missing | 204 (60.2%)  37 (10.9%)  98 (28.9%) | 186 (59.0%)  46 (14.6%)  83 (26.3%) | 390 (59.6%)  83 (12.7%)  181 (27.7%) | 0.201 |
| **Diagnosis**  F00-F09 (dementia)  F10-F19 (drug/alcohol)  F20-F29 (SSD)**  F30-F39 (mood)  F40-F48 (anxiety)  F50-F59 (eating disorders)  F60-F60 (PD)***  F90-F98 (childhood onset)  Missing any diagnosis | 22 (6.5%)  37 (14.7%)  120 (47.8%)  57 (22.7%)  28 (11.2%)  2 (0.8%)  39 (15.5%)  11 (4.4%)  138 (40.7%) | 8 (2.5%)  15 (5.4%)  50 (18.1%)  57 (20.6%)  69 (24.9%)  4 (1.4%)  37 (13.4%)  29 (10.5%)  120 (38.1%) | 30 (4.6%)  52 (9.9%)  170 (32.2%)  114 (21.6%)  97 (18.4%)  6 (1.1%)  76 (14.4%)  40 (7.6%)  258 (39.4%) | 0.014  <0.001  <0.001  0.552  <0.001  0.690  0.498  0.008 |
| **Antipsychotic** (ever prescribed)  Yes  No | 230 (67.9%)  109 (32.2%) | 112 (35.6%)  203 (64.4%) | 312 (47.7%)  342 (52.3%) | <0.001 |
| **Other psychotropic** (ever prescribed)  Yes  No | 254 (74.9%)  85 (25.1%) | 181 (57.5%)  134 (42.5%) | 435 (66.5%)  219 (33.5%) | <0.001 |
| **Admitted to hospital before cohort entry**  Yes  No | 65 (19.2%)  274 (80.8%) | 25 (7.9%)  290 (92.1%) | 90 (13.8%)  564 (86.2%) | <0.001 |
| **Cohort contribution years** (mean, SD) | 8.28 (1.53) | 7.57 (2.30) | 7.94 (1.97) | <0.001 |

**Supplementary figure 1 Time to readmission or crisis team contact after first admission in cohort period**


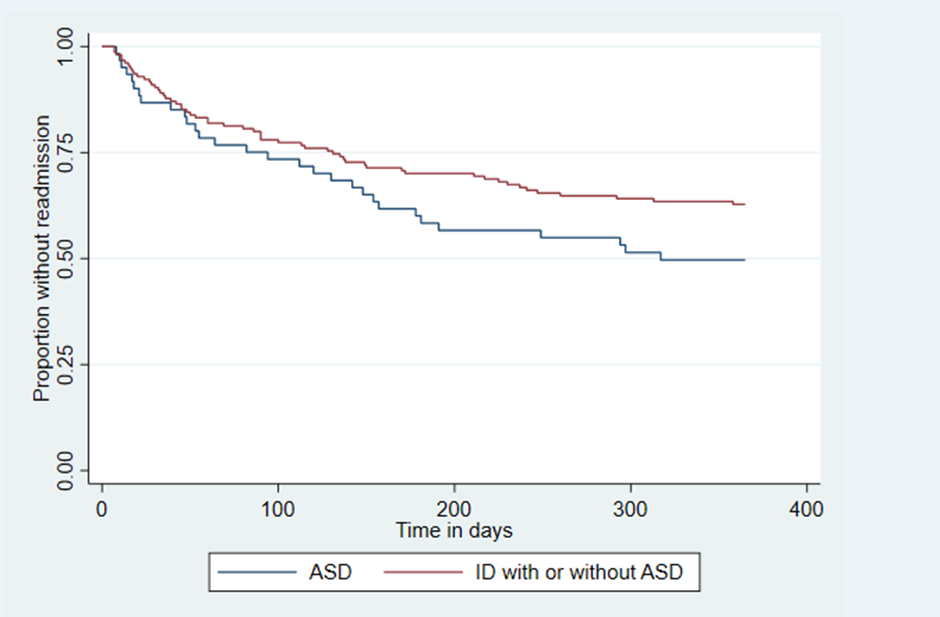

Supplement: Supplementary file 1 [file S2056472420001350sup001.docx]
